# Cumulative Dosage of Intrathecal Chemotherapy Agents Predicts White Matter Integrity in Long-Term Survivors of Acute Lymphoblastic Leukemia: A PETALE Study

**DOI:** 10.3390/cancers16061208

**Published:** 2024-03-19

**Authors:** Julie Laniel, Serge Sultan, Daniel Sinnett, Caroline Laverdière, Maja Krajinovic, Philippe Robaey, Luc Duong, Sarah Lippé

**Affiliations:** 1 Sainte-Justine University Health Center (SJUHC), Montreal, QC H3T 1C5, Canadasarah.lippe@umontreal.ca (S.L.); 2 Department of Psychology, Université de Montréal, Montreal, QC H2V 2S9, Canada; 3 Department of Pediatrics, Université de Montréal, Montreal, QC H3T 1C5, Canada; 4 Department of Pharmacology, Université de Montréal, Montreal, QC H3C 3J7, Canada; 5 Children’s Hospital of Eastern Ontario (CHEO), Ottawa, ON K1H 8L1, Canada; 6 Department of Psychiatry, Université de Montréal, Montreal, QC H3T 1J4, Canada; 7 Department of Psychiatry, University of Ottawa, Ottawa, ON K1N 6N5, Canada; 8 Department of Software Engineering and Information Technology, École de Technologie Supérieure (ETS), Montreal, QC H3C 1K3, Canada

**Keywords:** acute lymphoblastic leukemia, pediatric cancer, long-term survivors, neuropsychology, neurocognitive sequelae, magnetic resonance imaging

## Abstract

**Simple Summary:**

Acute lymphoblastic leukemia (ALL) is the most common childhood cancer in North America, with a survival rate of 85%. Despite improved outcomes, many survivors experience long-term side effects, including cognitive issues. This study investigates whether a brain imaging technique called Magnetization Transfer Ratio (MTR) can detect changes in the brain’s white matter of ALL survivors and if these changes are related to cognitive problems. Lower MTR levels in survivors, indicating myelin damage, correlated with cognitive impairments. Additionally, a link between MTR levels and the doses of certain chemotherapy drugs received was discovered. These findings improve our understanding of ALL survivorship and highlight the importance of MTR in monitoring brain health during and after cancer treatment.

**Abstract:**

Acute lymphoblastic leukemia (ALL) stands as the most prevalent form of pediatric cancer in North America, with a current five-year survival rate of 85%. While more children achieved ALL remission and transition into adulthood, the prevalence of long-term treatment-related effects, especially neurocognitive sequelae, remains significant. This study pursues two objectives. Firstly, it investigates if Magnetization Transfer Ratio (MTR), a method assessing myelin integrity, is sensitive to white matter (WM) microstructural changes in long-term ALL survivors and whether these relate to cognitive impairments. Secondly, it examines the dose-related effects of chemotherapy agents on the MTR and its relationship to other risk factors such as female sex, early age diagnosis, and cranial radiotherapy. Magnetization transfer imaging was utilized to assess WM integrity in 35 survivors at a mean of 18.9 years after the onset of ALL (range since diagnosis: 6.9–26.8). Additionally, 21 controls matched for age, sex, and education level, with no history of cancer, were included. MTR was extracted from both the entire brain’s WM and the corpus callosum through semi-automated procedures. The results indicated lower MTR means in survivors, which is linked to cognitive function. Negative associations between MTR means and intrathecal agents’ (MTX, cytarabine, and hydrocortisone) cumulative doses received were highlighted. This study offers valuable insights into the connections between myelin deterioration, cognitive impairment, and the implications of IT chemotherapy, enhancing our understanding of ALL survivorship dynamics. It underscores MTR’s relevance in monitoring neurotoxicity during oncological drug follow-up examinations.

## 1. Introduction

Acute lymphoblastic leukemia (ALL) remains the most diagnosed pediatric cancer. Fortunately, current treatments allow a 5-year survival rate as high as 80–90% [1]. Treatment regimens are based on a combination of chemotherapeutic agents directed to the central nervous system by intravenous and intrathecal routes and adjunctive cranial radiotherapy when the risk of relapse is critical. Due to the high risk of cognitive sequelae, cranial radiotherapy is increasingly being replaced by intensified systemic and intrathecal (IT) therapy [1,2,3]. Over a period of about 2 years, the child diagnosed with ALL receives aggressive treatments, which, given at a time when major developmental changes are taking place, can disrupt brain development [4]. Therefore, treatments with or without irradiation are associated with long-term neurocognitive sequelae in survivors expressed by reduced scores on the neuropsychological assessment of intellectual and executive functioning [5,6,7], as well as damage to brain tissue, mainly white matter (WM) [8,9,10]. Several risk factors contribute to the development of neurocognitive complications: younger age at diagnosis, female sex, cranial radiotherapy (CRT), and overall treatment intensity [11,12,13,14].

Part of the neurotoxicity of oncological treatments results in demyelination of the WM [15]. Particularly in children, newly synthesized myelin is even more vulnerable due to its higher metabolic activity and lower stability [4]. The emergence of late neurocognitive impairments is thought to be influenced by the level of premorbid brain integrity and the extent of neurotoxic effects, which involve direct WM impairments as well as disruption of mechanisms that facilitate tissue remyelination and compensatory processes [16,17]. As a result, survivors of ALL often show neurocognitive impairments such as WM volume loss and disrupted WM integrity [18]. Various methods have been employed to capture WM abnormalities in long-term ALL survivors’ brains. According to Wu et al., (2012) [19], magnetization transfer ratio (MTR) is highly sensitive to myelin content and axonal density and can detect subtle brain abnormalities that are not apparent in conventional magnetic resonance imaging techniques. The extent to which the WM microstructural integrity at adult age determines the ensuing cognitive functioning of ALL survivors remains unclear.

According to the foregoing, the aim of this study is to investigate WM structural and microstructural integrity in long-term ALL survivors using volumetric investigation and magnetization transfer imaging. WM volumes and MTR are investigated, on the one hand, in relation to neuropsychological outcomes and, on the other hand, in relation to neurocognitive risk factors (i.e., age at diagnosis, sex, and adjunctive CRT), cumulative doses of corticosteroids (i.e., prednisone, hydrocortisone, and dexamethasone) and intrathecal chemotherapy agents (i.e., IT-cytarabine, IT-methotrexate, IT-hydrocortisone). In addition to whole-brain measurements, particular interest was carried toward the corpus callosum (CC). The CC is the largest WM fiber bundle connecting the two cerebral hemispheres. The integrity of the interhemispheric connection pathways is essential for the proper functioning of the brain. Several studies observed microstructural changes in the CC following oncological treatments based on systemic chemotherapy [20,21,22,23,24]. However, very few studies have focused on the MTR. This study will enable a comprehensive examination of dose-related effects on magnetization transfer measures. We hypothesized reduced WM volume and MTR means in long-term ALL survivors compared to the healthy control group. Associations between neuroimaging measures, cumulative doses of chemotherapy agents, and cognitive performance indices are expected. More specifically, we hypothesize that the MTR may reflect neurotoxic damage in ALL survivors.

## 2. Methods

### 2.1. Study Design and Recruitment

This retrospective study is part of the PETALE research program at Sainte-Justine University Health Center (SJUHC), Quebec, Canada, which was designed to identify and characterize ALL long-term complication biomarkers. As described in Marcoux et al., (2017) [25], the PETALE cohort is composed of 246 ALLs diagnosed between age 0 and age 17, treated with the Dana Farber Cancer Institute protocols 87-01 to 05-01, and at least 5 years post-diagnosis at the data collection time point, without any history of refractory ALL, disease recurrence, or hematopoietic stem cell transplantation. According to their performance at the DIVERGT screening procedure [5,26], a sample of 35 ALL survivors (age range (17–40)), aiming to represent all spectrum of cognitive performance found in the initial cohort, was selected, tested using anatomical MRI studies and included in this study. For comparison purposes, 21 age- and education-level-matched healthy controls (age range (19–36)), representative with respect to sex, with no history of neurological, psychological, or cancer disorders, were recruited within SJUHC Research Center and within social networks. The complete recruitment procedure is detailed elsewhere [27]. The study was approved by the Institutional Review Board of SJUHC, and investigations were carried out in accordance with the principles of the Declaration of Helsinki. Written informed consent was obtained from study participants or parents/guardians.

### 2.2. Data Collection and Study Procedures

#### 2.2.1. Neuroimaging Protocol

MRI was performed on a General Electric Discovery MR750 3 Tesla system at SJUHC. MT spoiled gradient echo (MT-SPGR) [28] was used as acquisition method for magnetization transfer imaging by means of the following imaging sequences: 3D T_1_-weighted inversion–recovery magnetization prepared–ultrafast acquisition gradient echo (IR-FSPGR) [repetition time (TR)/echo (TE): 8.16/3.18 ms, inversion time (TI): 450 ms, matrix: 256 × 256 × 188, field of view (FOV): 0.75 × 0.75 × 1.5 mm and flip angle: 9°], 3D SPGR (MT saturation pulse off) and 3D MT-SPGR (MT saturation pulse on) [TR/TE: 32/4 ms, matrix: 256 × 256 × 104, FOV: 0.75 × 0.75 × 1.5 mm and flip angle: 10°].

#### 2.2.2. Neuroimaging Postprocessing

Images postprocessing was conducted using the FreeSurfer Software Suite v6 [29] (http://surfer.nmr.mgh.harvard.edu/, accessed on 20 June 2022). Each participant’s MRI data were processed independently to produce one mask per participant. This cortical reconstruction pipeline includes non-parametric, non-uniform intensity normalization, automated Talairach transformation, skull-stripping [30], segmentation of the subcortical white matter [31], intensity normalization [32], tessellation, surface smoothing, inflation, quasi-homeomorphic spherical transformation, and automated topology correction [33]. As a result, 256 axial slices without gaps covering the entire brain were obtained from both 3D SPGR and 3D SPGR-MT, thereby acquiring an unsaturated data set and a saturated data set. Additionally, segmentation masks were generated from the 3D T1 IR-FSPGR evenly processed data set. The segmentation of WM volumes of interest (VOIs) implied automated and customized procedures. The segmentation of the whole brain subcortical WM and its right and left hemispheres’ parcellation has been efficiently executed through FreeSurfer’s automated process, although the CC segmentation required manual correction due to the undesired inclusion of neighboring voxels, mostly from the fornix. CC was divided into five equal sections along the length, enabling interhemispheric communication that supports distinct cognitive functions—anterior, mid-anterior, central, mid-posterior, and posterior sections correspond to the rostrum, genu, body, isthmus, anterior splenium, and posterior splenium, respectively. Intracranial volume and cerebral WM volume in mm^3^ were computed with FreeSurfer. Intracranial volume was estimated using an atlas normalization procedure [34].

#### 2.2.3. Magnetization Transfer Processing

To generate MTR data, each participant’s MRI data were processed independently with an FSL pipeline (FMRIB Software v5.0). Images co-registration was performed using the FLIRT [35] linear registration algorithm. Since SPGR data featured a higher defined contrast, it has been selected as reference images. Hence, MT-SPGR data were normalized to SPGR data to create a reference volume, which was then spatially co-registered to the whole head high-resolution T1-weighted IR-FSPGR. To ensure a proper comparison, an optimized registration procedure involving a rigid body transformation with 6 degrees of freedom was used. The resulting co-registered images were used to calculate the MTR maps voxel-by-voxel via the fslmaths program according to the following formula: MTR = (SPGR − (SPGR-MT))/SPGR. To extract MTR data from each VOI, a simple intersection between the 3D MTR data maps and the segmentation masks was possible since the MTR data volume and all MRI images of the same participant had been previously recalibrated and standardized. The corresponding voxels were intersected with the VOIs’ coordinates in the segmentation masks. An outlier’s correction fixed at ±1 standard deviation (SD) was then applied to our voxel-wise MTR data set to avoid potential errors affecting the MTR mean in CC VOIs due to imperfections in the CC segmentation and parcellation. Mean MTR was computed and defined as the average MTR of all voxels in each 3D VOI: whole brain WM, left hemisphere WM, right hemisphere WM, whole CC, anterior CC, mid-anterior CC, mid-posterior CC, central CC, posterior CC.

#### 2.2.4. Cognitive Assessment

All participants enrolled in the study were evaluated using a set of neuropsychological tests covering intellectual and executive functioning. The neuropsychological evaluation was conducted by a qualified examiner through a standardized testing protocol that is already detailed in Boulet-Craig et al., (2018) [5]. Intellectual functioning was assessed with the 10 core subtests of the Wechsler Adult Intelligence Scale 4th edition (WAIS-IV) [36]. Age-adjusted scores of the domain-specific WAIS-IV subtests were summarized and transformed into the four WAIS-IV indexes (i.e., Verbal Comprehension Index (VCI), Perceptual Reasoning Index (PRI), Working Memory Index (WMI), Processing Speed Index (PSI)) along with the Full-Scale IQ (FSIQ) and the General Ability Index (GAI). The FSIQ encompasses overall intellectual functioning, while the GAI focuses specifically on reasoning abilities. The WAIS-IV index scores, in addition to the FSIQ and the GAI, are standardized to a mean of 100, with one standard deviation reflected in 15-point increments. Executive functioning was assessed with a DIVERGT [26] equivalent battery, including Digit Span [36], Verbal Fluency subtests [37], Trail Making Test [37], and Grooved Pegboard Test [38]. Raw scores were converted to age-adjusted scaled scores (mean [M] = 10, standard deviation [SD] = 3) based on nationally representative normative data. With the intention of quantifying the extent of ALL-associated cognitive sequelae on a singular composite score reflecting the most common executive deficits following ALL (i.e., working memory, verbal fluency, cognitive flexibility, and visuomotor processing speed), a global index of executive functioning was calculated for each participant. This Executive Functioning Index was obtained by computing the arithmetic mean of the following subtests’ scaled scores: Digit Span, Verbal Fluency—Condition 1, Trail Making Test—Condition 4, and Grooved Pegboard—Dominant Hand.

### 2.3. Statistical Analysis

All analyses were carried out using IBM SPSS statistics 28. Initial group comparisons were conducted to ensure that ALL survivors and controls were matched on key demographic factors. Fischer’s exact test was used to compare groups’ sex ratios. An independent sample *t*-test and its nonparametric equivalent Mann–Whitney U test were respectively run to test for differences between groups in age at assessment and number of years of education.

Comparison of neuropsychological test results and neuroimaging outcomes between ALL survivors and controls were made using independent samples *t*-tests or Mann–Whitney U for non-normally distributed variables. Test results were examined for effect sizes using Pearson’s correlation coefficient r. The magnitude of the observed effect was considered small when r varied around 0.10, medium when r varied around 0.30, and large if r reached 0.50 [39].

To examine the relationship between neuropsychological outcomes and cumulative dose of the chemotherapy agents, we employed directional Pearson correlations, shedding light on potential dose-response effects. Neuroimaging outcomes were also examined for associations with cognitive function using directional Pearson correlations, and their covariation with the cumulative doses of chemotherapy agents. Where applicable, both survivors and controls were included in correlational analyses.

To further explore the potential influence of the female sex as a risk factor, we conducted additional two-way ANOVAs to investigate sex-group interactions, specifically assessing their impact on cognitive and neuroimaging outcomes. ANOVAs’ effect sizes were assessed using partial η^2^ (η^2^ partial), where 0.01, 0.06, and 0.014 correspond to small, medium, and large effect sizes [40].

Finally, hierarchical multiple regression analyses were carried out to study the relative contribution of the cumulative dose of chemotherapy agents among the risk factors (i.e., female sex, early age at diagnostic, and adjunctive CRT) in predicting MTR metrics in the brain at adulthood. Regression models were adjusted for current age. Interactions between dose and sex and between dose and age at diagnosis were also explored. Effects sizes were interpreted using R2 as a percentage of variance explained, where 1%, 9%, and 25%, respectively, indicated small, medium, and large effects [40].

Given the well-documented neurocognitive sequelae in ALL survivors and our a priori hypothesis regarding their overall lower brain integrity compared to healthy controls, we conducted one-tailed tests. Test results were examined for statistical significance (*p* ≤ 0.05). To be aware of type 1 errors, the Benjamini–Hochberg false discovery rate (FDR) procedure [41,42] was applied for multiple comparisons [43]. In correlational analyses, the FDR correction was applied on a dependent variable-by-dependent variable basis. Considering that the correction for multiplicity may increase the risk of type 2 errors [44], uncorrected *p*-values are reported throughout the manuscript, and FDR-adjusted *p*-values (FDR adj.-*p*) are also provided where appropriate. The FDR threshold was fixed at 0.05.

## 3. Results

Demographic variables and treatment characteristics are displayed in Table 1. ALL survivors were assessed in neuropsychology and neuroimaging for the present study on average at 18.90 ± 5.37 years post-diagnosis and are therefore considered very long-term survivors. Neuropsychological outcomes are presented in Table 2. ALL survivors did not differ from controls either on the working memory index (WMI) (*p* = 0.623) or on the perceptual reasoning index (PRI) (*p* = 0.132). However, in comparison to the control group, ALL survivors exhibit lower average scores for the full-scale IQ (FSIQ) (*p* = 0.008, FDR adj.-*p* = 0.022), the general ability index (GAI) (*p* = 0.013, FDR adj.-*p* = 0.024), the verbal comprehension index (VCI) (*p* = 0.008, FDR adj.-*p* = 0.022), and the processing speed index (PSI) (*p* = 0.003, FDR adj.-*p* = 0.022). On the Executive Functioning Index based on DIVERGT scores, survivors underperformed compared to controls (*p* = 0.012, FDR adj.-*p* = 0.024), suggesting relative weakness in executive functioning. ALL survivors’ scores were inferior to controls on the Trail making test condition 4 (*p* = 0.016, FDR adj.-*p* = 0.022) and on the Grooved pegboard (*p* = 0.005, FDR adj.-*p* = 0.022), which may reflect more specific executive weaknesses in cognitive flexibility and visuomotor processing speed. ALL survivors’ scores on the Digit span (*p* = 0.465) and on the Verbal fluency condition 1 (*p* = 0.248) did not differ from those of the controls.

Table 3 presents the correlation analyses between the cumulative doses of chemotherapy agents received during treatments and the neuropsychological outcomes in adulthood. At first sight, IT-MTX stands out through the substantial correlations observed between its cumulative dose and several indices of cognitive performance (i.e., FSIQ, GAI, PRI, WMI, EF index), suggesting a higher dose of IT-MTX is associated with poorer general intellectual abilities, working memory and executive functioning. Nonetheless, the FDR correction led to the loss of statistical significance for the associations with the WMI and EF index. Additionally, we found moderate to strong negative correlations between the total IT-cytarabine dose and FSIQ, GAI, and PRI. After considering the multiplicity correction, these results still demonstrated statistical significance. In contrast to the other IT agents, no significant correlations were observed between IT-hydrocortisone dosage and neuropsychological outcomes. Additionally, no evidence of associations was observed between the intravenous MTX dose or the effective corticosteroids dose and the neuropsychological measures.

Brain volume outcomes are provided in Table S1 (Supplementary Materials). ALL survivors evidenced a 6.7% smaller WM volume (t(54) = −1.87, *p* = 0.034, FDR adj.-*p* = 0.057, *r* = 0.25) as well as a 5.3% smaller intracranial volume (t(54) = −1.81, *p* = 0.038, FDR adj.-*p* = 0.057, *r* = 0.24) than controls. Intracranial volume is known as a proxy of the maximal brain volume attained following development. Therefore, comparisons were also made for WM volume fraction, which is the ratio between cerebral WM volume and intracranial volume. WM volume fraction was 1.4% smaller in ALL survivors compared to controls (t(54) = −0.78, *p* = 0.219, FDR adj.-*p* = 0.219, *r* = 0.11). Thereby, the WM volume difference was not statistically significant after adjusting for intracranial volume. Otherwise, the intracranial volume was not associated with age at the MRI time-point in ALL survivors and controls combined, and neither was the WM volume. In an interesting way, there was a trend between younger age at diagnosis and smaller intracranial volume (*r* = 0.266, *p* = 0.061).

Table S2 (Supplementary Materials) presents the magnetization transfer imaging outcomes. ALL survivors tend to exhibit lower MTR means compared to controls. The group differences (ALL survivors < controls) reached the threshold of statistical significance in the whole brain (t(54) = −1.74, *p* = 0.044, FDR adj.-*p* = 0.097, *r* = 0.23), the right hemisphere (t(54) = −1.74, *p* = 0.044, FDR adj.-*p* = 0.097, *r* = 0.23), and the left hemisphere (t(54) = −1.67, *p* = 0.050, FDR adj.-*p* = 0.097, *r* = 0.22), yet the multiplicity correction rendered the findings statistically inconclusive. In addition, interestingly, differences in MTR means (ALL survivors < controls) were on the borderline of statistical significance for two sections of the CC, the central section (U = 272, z = −1.62, *p* = 0.054, FDR adj.-*p* = 0.097, *r* = −0.22) and the mid-posterior section (t(54) = −1.67, *p* = 0.051, FDR adj.-*p* = 0.097, *r* = 0.22). The central and mid-posterior sections of the CC cover the body, the isthmus, and the anterior splenium, carrying fibers connecting the motor and premotor cortex, sensory cortex, association cortex, and visual areas [45,46]. These interhemispheric connections support motor planning, initiation and coordination, multimodal sensory processing, visual integration, and higher-order cognitive functions such as memory, language, and problem-solving [47,48,49,50,51]. Microstructural damage in these regions of the CC could possibly contribute to the cognitive weaknesses observed in ALL survivors, especially visuomotor coordination and processing speed. Nevertheless, our study did not yield a statistically significant difference in callosal regions’ mean MTR, potentially attributable to limited statistical power.

Regarding the female sex risk factor, separate two-way ANOVAs were conducted to test for sex-group interactions, with neuropsychological and neuroimaging outcomes as dependent variables. No sex-group interaction was found to be significant. No main effect of sex was found on the neuropsychological outcomes. However, we observed a main effect of sex (females < males) on the WM volume (uncorrected for intracranial volume) (F = 21.23, η^2^_partial_ = 0.290, *p* < 0.001, FDR adj.-*p* = 0.001), the intracranial volume (F = 20.51, η^2^_partial_ = 0.283, *p* < 0.001, FDR adj.-*p* = 0.001), and the CC mean MTR (F = 8.23, η^2^_partial_ = 0.136, *p* = 0.006, FDR adj.-*p* = 0.018). Otherwise, no main effect of sex was found on the whole brain mean MTR (*p* = 0.290, FDR adj.-*p* = 0.290) nor on the WM volume fraction (*p* = 0.075, FDR adj.-*p* = 0.092). Indeed, men generally have larger head sizes and tend to exhibit larger brain volume in comparison to women [52,53,54]. Thus, sex differences in WM volume tend to disappear when considering intracranial volume. Further, an effect of sex on the MTR of the corpus callosum has been reported in normal adults; however, the literature remains inconsistent, with some studies suggesting that males exhibit higher callosal MTR values than females. For example, Björnholm and colleagues (2017) [55] found sex-related differences in all sections of the CC among 433 all-comer adults (mean age = 26.50, SD = 0.51). However, other studies have found none [56,57].

Correlation analyses between neuroimaging outcomes and neuropsychological measures are presented in Table S3 (Supplementary Materials). Without adjustment for intracranial volume, the MW volume was correlated to FSIQ (*r* = 0.227, *p* = 0.046, FDR adj.-*p* = 0.108), GAI (*r* = 0.368, *p* = 0.003, FDR adj.-*p* = 0.009), VCI (r = 0.355, *p* = 0.004, FDR adj.-*p* = 0.012), and PRI (*r* = 0.246, *p* = 0.034, FDR adj.-*p* = 0.075). After the FDR correction for multiple comparisons, only the associations with GAI and VCI remained significant. The WM volume fraction was correlated to GAI (*r* = 239, *p* = 0.038, FDR adj.-*p* = 0.038) and VCI (*r* = 0.232, *p* = 0.043, FDR adj.-*p* = 0.043), and the findings were unaffected by the FDR correction.

Moreover, the whole brain mean MTR was associated with GAI (*r* = 0.227, *p* = 0.046, FDR adj.-*p* = 0.067) and VCI (*r* = 0.239, *p* = 0.038, FDR adj.-*p* = 0.057). The right hemisphere MTR mean was associated to FSIQ (*r* = 0.234, *p* = 0.041, FDR adj.-*p* = 0.115), GAI (*r* = 0.241, *p* = 0.037, FDR adj.-*p* = 0.067), VCI (*r* = 0.240, *p* = 0.037, FDR adj.-*p* = 0.057), and PSI (*r* = 0.260, *p* = 0.029, FDR adj.-*p* = 0.125). The left hemisphere MTR mean was associated with VCI (*r* = 0.228, *p* = 0.046, FDR adj.-*p* = 0.059). The mean MTR in the CC was correlated with GAI (*r* = 0.259, *p* = 0.027, FDR adj.-*p* = 0.067) and VCI (*r* = 0.271, *p* = 0.022, FDR adj.-*p* = 0.057). Several statistically significant correlations were also found for different callosal sections. The mean MTR in the anterior section correlated with GAI (*r* = 0.228, *p* = 0.045, FDR adj.-*p* = 0.451). The mean MTR in the mid-anterior section correlated with FSIQ (*r* = 0.244, *p* = 0.035, FDR adj.-*p* = 0.115), GAI (*r* = 0.282, *p* = 0.018, FDR adj.-*p* = 0.067), VCI (*r* = 0.243, *p* = 0.035, FDR adj.-*p* = 0.057), and PRI (*r* = 0.249, *p* = 0.032, FDR adj.-*p* = 0.239). The mean MTR in the mid-posterior section correlated with FSIQ (*r* = 0.232, *p* = 0.043, FDR adj.-*p* = 0.115), GAI (*r* = 0.238, *p* = 0.039, FDR adj.-*p* = 0.067) and VCI (*r* = 0.278, *p* = 0.019, FDR adj.-*p* = 0.057). The mean MTR in the posterior section correlated with VCI (*r* = 0.258, *p* = 0.027, FDR adj.-*p* = 0.057). On the other hand, all these associations with MTR means were no longer significant after the multiplicity correction.

**Figure 1:**
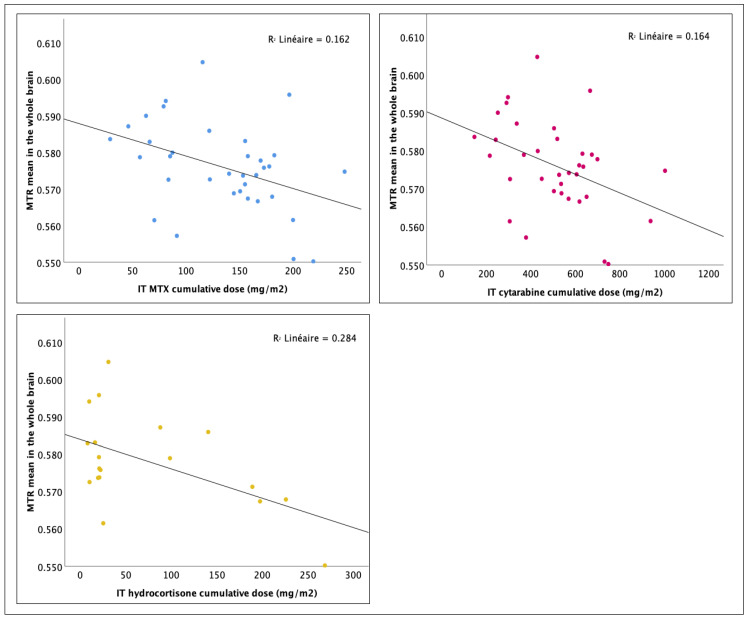
Effect of cumulative dosage of intrathecal chemotherapy agents on the whole brain mean MTR: linear regression analysis.

Correlation analyses between neuroimaging outcomes and chemotherapy agents’ cumulative doses are presented in Table 4. No association between WM volume and cumulative doses was found. The effective corticosteroid cumulative dose was associated with smaller intracranial volume in survivors (*r* = −0.298, *p* = 0.041, FDR adj.-*p* = 0.068), yet the multiplicity correction rendered those findings statistically inconclusive. No association was found between intravenously administered MTX dosage and the neuroimaging outcomes, suggesting less neurotoxicity from intravenous administration compared to IT administration. In agreement with the above, cumulative doses of intrathecally administered chemotherapy, including MTX, demonstrated substantial negative correlations with the MTR means. Higher IT-MTX dose was associated with a smaller mean MTR in the whole brain (*r* = −0.403, *p* = 0.008, FDR adj.-*p* = 0.015), the right hemisphere (*r* = −0.434, *p* = 0.005, FDR adj.-*p* = 0.013), the left hemisphere (*r* = −0.347, *p* = 0.021, FDR adj.-*p* = 0.035), the whole CC (*r* = −0.283, *p* = 0.050, FDR adj.-*p* = 0.083), and its anterior section (*r* = −0.351, *p* = 0.019, FDR adj.-*p* = 0.048). The correlations between IT-MTX dosages and MTR means maintained their statistical significance after the FDR correction except for the whole CC. Similar results have been found for IT-cytarabine. The IT-cytarabine cumulative dose was negatively correlated with the mean MTR in the whole brain (*r* = −0.405, *p* = 0.008, FDR adj.-*p* = 0.015), the right hemisphere (*r* = −0.437, *p* = 0.004, FDR adj.-*p* = 0.013), the left hemisphere (*r* = −0.349, *p* = 0.020, FDR adj.-*p* = 0.035), the whole CC (*r* = −0.284, *p* = 0.049, FDR adj.-*p* = 0.083), and its anterior section (*r* = −0.359, *p* = 0.017, FDR adj.-*p* = 0.048). These associations survived correction for multiple comparisons except for the whole CC. As IT-MTX and IT-cytarabine dosages were strongly related to each other in our sample (*r* = 0.954, *p* < 001), it is unclear whether neurotoxic effects arise more from one IT agent or the other or from both. The literature extensively documents MTX’s long-term neurotoxicity, ranking it among the most neurotoxic chemotherapy agents, while recent findings raise concerns about a potentially harmful interaction between IT-MTX and IT-cytarabine [58,59,60,61,62]. Furthermore, the IT-hydrocortisone cumulative dose was negatively correlated with the mean MTR in the whole brain (*r* = −0.533, *p* = 0.009, FDR adj.-*p* = 0.015), the right hemisphere (*r* = −0.532, *p* = 0.009, FDR adj.-*p* = 0.015), the left hemisphere (*r* = −0.512, *p* = 0.013, FDR adj.-*p* = 0.035), the whole CC (*r* = −0.398, *p* = 0.046, FDR adj.-*p* = 0.083), and its anterior section (*r* = −0.405, *p* = 0.043, FDR adj.-*p* = 0.072), mid-anterior section (*r* = −0.465, *p* = 0.022, FDR adj.-*p* = 0.110), and mid-posterior section (*r* = −0.393, *p* = 0.048, FDR adj.-*p* = 0.240). After FDR correction, the dosage associations with the mean MTR in the whole brain, the right hemisphere, and the left hemisphere maintained their statistical significance. Note that the IT-hydrocortisone dosages were not correlated with the IT-MTX (*p* = 0.286) and IT-cytarabine dosages (*p* = 0.320). Figure 1 displays the scatter diagrams of the relationship between IT agents’ dosage and the MTR mean in the whole brain.

Based on the previous results, each of the IT agents (i.e., IT-MTX, IT-cytarabine, IT-hydrocortisone) was included as an independent variable separately in the multiple regression models with the mean MTR in the whole brain, and the mean MTR in the CC as the dependent variables (six regression models). In the first step of the regression models, current age (continuous variable), age at diagnosis (continuous variable), sex (binary variable), and adjunctive CRT (binary variable) were introduced using an enter method. In the second step, the cumulative dose of the chemotherapeutic agent (i.e., IT-MTX, IT-cytarabine, IT-hydrocortisone) was introduced. In the models including IT-MTX as a predictor, in the context of a sensitivity analysis, the cumulative leucovorin dose was introduced in the next step because of its potential neuroprotective effect suggested by previous studies [13,60]. The purpose of this sensitivity analysis was to determine whether significant associations would be affected by the inclusion of the leucovorin dose as an additional factor. In the last step of all models, interactions between the dose of chemotherapy agents and sex, as well as the age at diagnosis, were explored by adding interaction terms. The sex variable was coded with a value of 1 for female sex and 0 for male sex. The radiotherapy variable was coded with a value of 1 for treatment including CRT and 0 for chemotherapy-only treatment. All continuous predictors included in the regression models were centered around their mean, resulting in a transformation that set their means to 0. Interaction terms were calculated from the mean-centered predictors. Thus, beta coefficients should be interpreted as the average change in the outcome variable for a one-unit change in the predictor from its mean value. Standardized beta coefficients indicate the change in the outcome variable associated with a one-standard-deviation change in the predictor, allowing for comparisons of the relative importance or strength of the predictors in influencing the outcome variable.

The regression models and associated statistics are presented in Table 5 and Tables S4–S8. In the first step of the models, controlling for current age, the combination of the risk factors (i.e., age at diagnosis, sex, and CRT) explained, in a non-significant way, 18% of the variance of the mean MTR in the whole brain (F_4,30_ = 1.623, *p* = 0.194), and in a significant way, 29% of the variance of the mean MTR in the CC (F_4,30_ = 2.987, *p* = 0.035). No covariates were significant in the models predicting the mean MTR in the whole brain. Sex was found to be a significant covariate of the mean MTR in the CC (B = −0.009, β = −0.366, *p* = 0.039), with female sex associated with a reduced callosal mean MTR. In the following step, separately, the cumulative dose of IT-MTX, IT-cytarabine, and IT-hydrocortisone, respectively, added a significant contribution of 16% (ΔF_1,29_ = 7.244, *p* = 0.012, β = −0.704), 15% (ΔF_1,29_ = 6.353, *p* = 0.017, β = −0.582), and 14% (ΔF_1,29_ = 5.917, *p* = 0.021, β = −0.696) to the prediction of the mean MTR in the whole brain, and 11% (ΔF_1,29_ = 5.228, *p* = 0.030, β = −0.574), 10% (ΔF_1,29_ = 4.536, *p* = 0.042, β = −0.471), and 11% (ΔF_1,29_ = 5.016, *p* = 0.033, β = −0.606) for the mean MTR in the CC. The inclusion of leucovorin did not impact the association between IT-MTX dose and the mean MTR in the whole brain or the CC. No dose interactions were found with sex or age at diagnosis.

## 4. Discussion

Over the past decades, advancements in therapeutic strategies have increased the survival rate of pediatric patients with ALL [63]. Despite these improvements, careful monitoring of neurocognitive development is crucial for survivors treated with MTX, as the drug poses a risk of both acute and chronic neurotoxicity [64]. The literature also provides insights into the neurotoxicity associated with cytarabine [65,66,67,68] and hydrocortisone [69,70] in the context of triple IT therapy, as well as other corticosteroids (dexamethasone, prednisolone, and prednisone) [71,72,73], which can penetrate the blood–brain barrier and access the central nervous system.

This study explored WM integrity in relation to neurotoxicity risk factors among adult survivors of pediatric ALL. The findings further solidify the established link between WM microstructural changes and IT-MTX exposure while also shedding light on the dose effects of other IT agents, cytarabine and hydrocortisone [59,74]. This study provides further evidence for the idea, which is well-supported in the existing literature that the extent of WM microstructural changes is contingent upon the level of exposure to intrathecal MTX. Additionally, this study highlights the dose effects of the other IT agents, cytarabine and hydrocortisone. The cumulative dose of the different intrathecal chemotherapy agents is a factor that aggravates the adverse consequences on the cognitive and cerebral development of children treated for ALL. Moreover, it provides compelling evidence that the mean MTR is a valuable biomarker of long-term neurotoxicity among ALL survivors.

To summarize the key findings of our study, we identified fairly strong negative associations between MTR and dosages of IT agents among long-term survivors of ALL. These findings suggest that MTR could serve as a sensitive indicator of WM microstructural alterations in this population. Furthermore, lower MTR in the whole brain and CC, along with reduced WM volume fraction, were associated with lower GAI, reflecting weaker reasoning abilities. Regression analysis, controlling for relevant factors such as current age, sex, age at diagnosis, and adjunctive cranial radiation therapy (CRT), confirmed the relationship between IT dosages and MTR in both the whole brain and CC. These results highlight the crucial role of MTR as a potential biomarker linking survivors’ cognitive complaints with treatment-induced neurotoxicity.

MTR reflects WM tissue composition, especially myelin content, and is sensitive to microstructural changes in myelin. Since in vitro studies, animal models, and post-mortem investigations collectively suggest that chemotherapy-induced neurotoxicity leads to demyelination [75], a decrease in MTR could be indicative of reduced myelination. While MTR can be influenced by various factors, including myelin integrity and axonal density, it is less sensitive to the spatial organization of WM tracts compared to Fractional Anisotropy (FA) [76]. FA is a more specific measure of the directionality and coherence of water diffusion along WM tracts, which reflects the spatial organization and alignment of WM fibers [77]. FA has been extensively investigated in ALL survivors, revealing decreased FA values in various regions, including the frontal lobe, the frontostriatal tracts, and the CC [24,78]. In contrast, very few studies have investigated MTR to detect WM alterations in ALL survivors. Yamamoto and coworkers (2006) [79] observed a decline in peak values within MTR histograms after MTX administration. On the other hand, a more recent study comparing magnetization transfer measures between ALL survivors and healthy controls ended with inconclusive results [15]. To the best of our knowledge, our study is the first to demonstrate the impact of cumulative doses of chemotherapy agents on MTR means.

In the ongoing quest for a neuroimaging measure sensitive to microstructural damage associated with chemotherapy-induced neurotoxicity, MTR emerges as a promising lead. We raise potential implications for both clinical practice and research. In clinical settings, where treatment-induced neurotoxicity is typically identified through neurological symptoms like seizures, implementing regular follow-up neuroimaging assessments using MTR could offer greatly improved monitoring of neurotoxicity. This heightened surveillance may facilitate earlier detection and enable treatment adjustments to be tailored more precisely according to the child’s individual response. In future research endeavors, the integration of MTR in imaging methodology could prove advantageous for exploring the cerebral and cognitive consequences of oncological treatments. Moreover, validation studies will provide valuable insights into the potential clinical implications of our findings and guide the development of more targeted interventions to mitigate neurotoxicity in cancer patients undergoing chemotherapy. The utilization of MTR may represent a compelling avenue for targeting the optimal dosages, aiming to achieve maximum efficacy while minimizing neurotoxicity and its ensuing consequences on the quality of life of cancer survivors.

Turning to another noteworthy observation, the difference in WM volume between ALL survivors and healthy controls did not remain after controlling for intracranial volume. We have not been able to demonstrate a volume loss specific to WM among ALL survivors in this way. As in some previous studies [9,80,81], a group difference was detected in intracranial volume, with survivors exhibiting a smaller intracranial volume compared to the control group. As the intracranial volume is an index of the global brain volume attained following development, it seems possible that the reduction in intracranial volume somehow reflects the disruption of normal brain development processes in the context of childhood ALL. While a relative loss of WM volume could not be demonstrated, we identified a significant decrease in the mean MTR throughout the whole brain among survivors compared to the control group and observed a trend in central-to-mid-posterior CC sections.

Our control group was matched for the level of education attained, the age at the time of the study, and sex. A common bias in studies of the neurocognitive status of ALL survivors is the control group, which tends to have an average IQ higher than the mean IQ of the normative population (100) [82]. Our recruitment efforts have allowed us to form a control group that has an average IQ of 104.9, which does not differ significantly from the normative population mean of 100 (*t*(20) = 1.622, *p* = 0.121). This achievement has contributed to our confidence in presenting the imaging results, as our groups show a considerable level of comparability. With the foregoing in mind, we have observed certain cognitive weaknesses in the group of ALL survivors, highlighting specific cognitive impairments associated with ALL treatments.

Our findings did not provide clear supporting evidence of sex having an impact on the degree of neurocognitive impairment in this cohort of survivors. A trend was observed toward lower MTR means in women compared to men, and a significant main effect of sex was found on the mean MTR of the CC. It is plausible that a reduction in MTR impacts women to a greater extent, given their lower MTR values compared to men. However, the present study did not investigate this hypothesis. Yet, evidence suggests that female sex carries an increased risk of neurocognitive impairment after ALL treatment. Multiple studies have identified sex-related differences in cognitive outcomes, revealing that female survivors tend to exhibit poorer cognitive functioning compared to their male counterparts [12,83,84,85]. Congruently, studies indicate a heightened susceptibility to structural and microstructural brain alterations in female survivors [4,73,78,86]. Girls have been shown to exhibit a smaller increase in WM during childhood compared to boys [87]. It is proposed that the variation in WM growth, along with hormonal differences, may render girls more susceptible to the neurotoxic effects of chemotherapy [88].

MTX is widely considered the primary culprit, although other agents may also contribute to neurotoxicity [89]. MTX-induced neurotoxicity arises from disruptions in folate physiology and homeostasis, which are vital for neuronal and central nervous system cell function, as they play critical roles in DNA and RNA synthesis, DNA methylation, and maintenance of myelin [60]. More broadly, several mechanisms have been proposed to explain the long-term neurocognitive damages resulting from ALL treatments based on high doses of chemotherapy. There is chemotherapy-induced suppression of cell proliferation, neuroinflammation, the loss of phospholipids affecting white matter architecture, and the disturbance of the developing neural networks in the immature brain [4,90]. In addition, other mechanisms that may have an additive indirect effect on the neurocognitive status of ALL survivors have been raised in the literature. For instance, ALL survivors are at increased risk for chronic cardiopulmonary conditions, which can impact cerebrovascular health by altering cerebral perfusion and blood oxygenation [91,92]. We are also listing metabolic and endocrine complications such as adrenal insufficiency (compromised hypothalamic–pituitary–adrenal function), hypogonadism, hypothyroidism, and growth hormone deficiency [91,93,94]. Systemic inflammation and oxidative stress are additionally highlighted [86,95,96,97,98]. In future studies exploring the long-term effects of chemotherapy agents on brain integrity and cognition, incorporating metabolic, oxidative, and inflammatory factors would be of great interest.

Limitations should be considered in the interpretation of these results. Our study had a relatively small sample size, which could have led to the analyses being underpowered. Replicating these findings with a larger cohort of survivors will be informative. Twenty-seven survivors out of the 35 included in this study received CRT. Of these, all received 18 Gy except one, which received 12 Gy. There is evidence to suggest that treatments combining chemotherapy and CRT are associated with greater brain volume loss and WM damage compared to chemotherapy-only treatments [99,100,101]. CRT is also known to increase the permeability of the blood–brain barrier, which could allow neurotoxic chemotherapy to penetrate the brain more effectively [102]. The combination of CRT and chemotherapy may be associated with greater neurotoxicity [64]. Therefore, the generalizability of the results to survivors treated only with chemotherapy is limited.

Limitations notwithstanding, our study provides sufficient indications that the MTR can capture the neurotoxic signature of intrathecal treatments almost two decades after pediatric ALL. Our results reveal a decrease in MTR in the whole brain WM and the CC in the adult brain as a function of the cumulative dose received of the IT agents, MTX, cytarabine, and hydrocortisone, during treatments.

## 5. Conclusions

In conclusion, this study elucidates the complex interplay between therapeutic interventions and long-term neurocognitive outcomes in survivors of pediatric acute lymphoblastic leukemia (ALL). Despite improved survival rates, ALL survivors are susceptible to neurotoxicity, particularly associated with methotrexate (MTX), cytarabine, and hydrocortisone treatments. The study underscores the significance of Magnetization Transfer Ratio (MTR) as a biomarker for assessing white matter integrity and cognitive impairments in ALL survivors. Notably, lower MTR levels were correlated with cumulative doses of intrathecal chemotherapy agents, highlighting the importance of personalized treatment approaches. While limitations exist, including sample size and the impact of cranial radiotherapy, these findings emphasize the need for continued research into mitigating neurotoxicity in ALL survivors and optimizing long-term outcomes. Integrating metabolic, oxidative, and inflammatory factors in future studies could provide further insights into the multifaceted nature of neurocognitive sequelae in this population. 

## Figures and Tables

**Table 1:** Demographics and clinical information.

	ALL Survivors (*n* = 35)	Controls (*n* = 21)	*p*
Demographics			
Sex, *n* (%)			
Male	21 (60)	12 (57.1)	1.00 ^c^
Female	14 (40)	9 (42.9)	
Age at assessment	26.27 (6.39)	27.1 (4.7)	0.620 ^d^
Years of education	12.63 (2.18)	15.00 [11.00–18.00] ^b^	0.080 ^e^
Treatment characteristics			
Age at diagnosis ^a^	7.37 (5.55)	N/A	-
DFCI protocol, *n* (%)			
87-01	5 (14.3)	N/A	-
91-01	11 (31.4)	N/A	-
95-01	13 (37.1)	N/A	-
00-01	3 (8.6)	N/A	-
05-01	3 (8.6)	N/A	-
Cranial radiation therapy, *n* (%)			
Yes *	27 (71.1)	N/A	-
No	8 (22.9)	N/A	-
Chemotherapy cumulative doses			
IT methotrexate (MTX) (mg/m^2^)	134.35 (54.42) ^a^	N/A	-
IT cytarabine (mg/m^2^)	513.11 (197.65) ^a^	N/A	-
IT hydrocortisone (mg/m^2^)	22.39 [8.20–268.67] ^b^	N/A	-
IV methotrexate (MTX) (mg/m^2^)	6042.06 [1777.47–12,750.46] ^b^	N/A	-
Effective corticosteroids dose (g/m^2^)	12,399.69 (5079.56) ^a^	N/A	

^a^ Mean (Standard deviation); ^b^ Median [Range]; ^c^ Fisher’s Exact Test; ^d^ Independent samples *t*-test; ^e^ Mann–Whitney U; DFCI: Dana Farber Cancer Institute; * Median [range], 18 Gy [12–18 Gy]; IV: intravenous; IT: intrathecal; N/A: Not applicable.

**Table 2:** Neuropsychological measures.

	ALLs (*n* = 35)	Controls (*n* = 21)	*p*	FDR adj.-*p*	Effect Size *r*
WAIS-IV scales					
FSIQ	94.14 (14.35) ^a^	104.9 (13.7) ^a^	**0.008 ^c^**	**0.022**	0.35
GAI	99.66 (11.81) ^a^	108.5 (13.5) ^a^	**0.013 ^c^**	**0.024**	0.33
VCI	98.23 (11.64) ^a^	111 (83–123) ^b^	**0.008 ^d^**	**0.022**	0.35
PRI	101.20 (14.57) ^a^	107.2 (13.8) ^a^	0.132 ^c^	0.182	0.20
WMI	94.40 (13.54) ^a^	94 (76–137) ^b^	0.623 ^d^	0.623	0.07
PSI	90.42 (20.63) ^a^	104.4 (12.7) ^a^	**0.003 ^c^**	**0.022**	0.39
DIVERGT scales					
Executive Functioning Index	8.70 (2.50) ^a^	10.10 (1.50) ^a^	**0.012 ^c^**	**0.024**	0.33
Digit span	7.00 [3.00–14.00] ^b^	8.86 (2.39) ^a^	0.465 ^d^	0.512	0.10
Verbal fluency condition 1	7.91 (3.02) ^a^	8.86 (2.74) ^a^	0.248 ^c^	0.303	0.16
Trail making test condition 4	10.00 [1.00–15.00] ^b^	11.00 [8.00–14.00] ^b^	**0.016 ^d^**	**0.022**	0.31
Grooved pegboard dominant hand	8.74 (3.36) ^a^	11.24 (2.59) ^a^	**0.005 ^c^**	**0.022**	0.37

^a^ Mean (Standard deviation); ^b^ Median [Range]; ^c^ Independent samples *t*-test; ^d^ Mann–Whitney U. Values in bold where *p* ≤ 0.05 (Two-tailed).

**Table 3:** Pearson’s *r* for correlations conducted between cumulative doses of chemotherapy agents and neuropsychological indices.

	FSIQ	GAI	VCI	PRI	WMI	PSI	EF Index
IT-MTX dose	−0.391 * *p* = 0.010 *p*_adj_ = 0.033	−0.387 * *p* = 0.011 *p*_adj_ = 0.027	−0.104 *p* = 0.276 *p*_adj_ = 0.380	−0.501 ** *p* = 0.001 *p*_adj_ = 0.005	−0.295 * *p* = 0.043 *p*_adj_ = 0.215	−0.250 *p* = 0.080 *p*_adj_ = 0.183	−0.305 * *p* = 0.037 *p*_adj_ = 0.125
IT-cytarabine dose	−0.375 * *p* = 0.013 *p*_adj_ = 0.033	−0.397 ** *p* = 0.009 *p*_adj_ = 0.027	−0.131 *p* = 0.226 *p*_adj_ = 0.380	−0.486 ** *p* = 0.002 *p*_adj_ = 0.005	−0.221 *p* = 0.101 *p*_adj_ = 0.253	−0.276 *p* = 0.060 *p*_adj_ = 0.183	−0.282 *p* = 0.050 *p*_adj_ = 0.125
IT-hydrocortisone dose	0.130 *p* = 0.479 *p*_adj_ = 0.479	−0.150 *p* = 0.476 *p*_adj_ = 0.476	−0.121 *p* = 0.311 *p*_adj_ = 0.380	−0.183 *p* = 0.226 *p*_adj_ = 0.377	−0.159 *p* = 0.258 *p*_adj_ = 0.323	0.134 *p* = 0.304 *p*_adj_ = 0.317	0.850 *p* = 0.365 *p*_adj_ = 0.426
IV-MTX dose	0.091 *p* = 0.302 *p*_adj_ = 0.378	0.027 *p* = 0.440 *p*_adj_ = 0.476	0.054 *p* = 0.380 *p*_adj_ = 0.380	−0.037 *p* = 0.417 *p*_adj_ = 0.417	0.153 *p* = 0.189 *p*_adj_ = 0.315	0.086 *p* = 0.317 *p*_adj_ = 0.317	0.033 *p* = 0.426 *p*_adj_ = 0.426
Effective corticosteroids dose	−0.156 *p* = 0.186 *p*_adj_ = 0.310	−0.132 *p* = 0.225 *p*_adj_ = 0.375	−0.153 *p* = 0.189 *p*_adj_ = 0.380	−0.066 *p* = 0.353 *p*_adj_ = 0.417	−0.025 *p* = 0.444 *p*_adj_ = 0.444	−0.219 *p* = 0.110 *p*_adj_ = 0.183	−0.169 *p* = 0.166 *p*_adj_ = 0.277

IT: intrathecal; IV: intravenous; *p*_adj_: FDR adjusted *p*-values. * Correlation is significant at the 0.05 level (One-tailed). ** Correlation is significant at the 0.01 level (One-tailed). Note: *p*-values adjusted for FDR separately for each dependent variable (neuropsychological indices).

**Table 4:** Pearson’s *r* for directional correlations conducted between neuroimaging outcomes and cumulative doses of chemotherapy agents.

	Effective Corticosteroids	IV MTX	IT MTX	IT Cytarabine	IT Hydrocortisone
MTR means					
Whole brain	0.090 *p* = 0.303 *p*_adj_ = 0.379	0.020 *p* = 0.445 *p*_adj_ = 0.445	−0.403 ** *p* = 0.008 *p*_adj_ = 0.015	−0.405 ** *p* = 0.008 *p*_adj_ = 0.015	−0.533 ** *p* = 0.009 *p*_adj_ = 0.015
Right hemisphere	0.079 *p* = 0.326 *p*_adj_ = 0.408	−0.034 *p* = 0.422 *p*_adj_ = 0.422	−0.434 ** *p* = 0.005 *p*_adj_ = 0.013	−0.437 ** *p* = 0.004 *p*_adj_ = 0.013	−0.532 ** *p* = 0.009 *p*_adj_ = 0.015
Left hemisphere	0.097 *p* = 0.290 *p*_adj_ = 0.301	0.091 *p* = 0.301 *p*_adj_ = 0.301	−0.347 * *p* = 0.021 *p*_adj_ = 0.035	−0.349 * *p* = 0.020 *p*_adj_ = 0.035	−0.512 * *p* = 0.013 *p*_adj_ = 0.035
Corpus callosum (CC)	0.009 *p* = 0.480 *p*_adj_ = 0.480	0.111 *p* = 0.263 *p*_adj_ = 0.329	−0.283 * *p* = 0.050 *p*_adj_ = 0.083	−0.284 * *p* = 0.049 *p*_adj_ = 0.083	−0.398 * *p* = 0.046 *p*_adj_ = 0.083
Anterior CC	−0.045 *p* = 0.399 *p*_adj_ = 0.399	0.059 *p* = 0.369 *p*_adj_ = 0.399	−0.351 * *p* = 0.019 *p*_adj_ = 0.048	−0.359 * *p* = 0.017 *p*_adj_ = 0.048	−0.405 * *p* = 0.043 *p*_adj_ = 0.072
Mid-anterior CC	0.084 *p* = 0.316 *p*_adj_ = 0.395	−0.011 *p* = 0.476 *p*_adj_ = 0.476	−0.241 *p* = 0.081 *p*_adj_ = 0.135	−0.241 *p* = 0.081 *p*_adj_ = 0.135	−0.465 * *p* = 0.022 *p*_adj_ = 0.110
Central CC	0.052 *p* = 0.384 *p*_adj_ = 0.499	0.256 *p* = 0.069 *p*_adj_ = 0.345	−0.019 *p* = 0.457 *p*_adj_ = 0.499	0.000 *p* = 0.499 *p*_adj_ = 0.499	−0.104 *p* = 0.336 *p*_adj_ = 0.499
Mid-posterior CC	−0.021 *p* = 0.453 *p*_adj_ = 0.453	0.196 *p* = 0.130 *p*_adj_ = 0.248	−0.148 *p* = 0.198 *p*_adj_ = 0.248	−0.148 *p* = 0.198 *p*_adj_ = 0.248	−0.393 * *p* = 0.048 *p*_adj_ = 0.240
Posterior CC	0.070 *p* = 0.345 *p*_adj_ = 0.411	0.039 *p* = 0.411 *p*_adj_ = 0.411	−0.260 *p* = 0.066 *p*_adj_ = 0.123	−0.250 *p* = 0.074 *p*_adj_ = 0.123	−0.365 *p* = 0.062 *p*_adj_ = 0.123
Volumes					
White matter (WM)	−0.178 *p* = 0.153 *p*_adj_ = 0.255	0.381 *p* = 0.012 *p*_adj_ = 0.060	−0.097 *p* = 0.290 *p*_adj_ = 0.290	−0.103 *p* = 0.278 *p*_adj_ = 0.290	0.227 *p* = 0.125 *p*_adj_ = 0.255
Intracranial volume	−0.298 * *p* = 0.041 *p*_adj_ = 0.068	0.358 *p* = 0.017 *p*_adj_ = 0.068	−0.135 *p* = 0.219 *p*_adj_ = 0.219	−0.142 *p* = 0.208 *p*_adj_ = 0.219	0.413 *p* = 0.039 *p*_adj_ = 0.068
WM volume fraction	0.169 *p* = 0.166 *p*_adj_ = 0.345	0.157 *p* = 0.184 *p*_adj_ = 0.345	0.014 *p* = 0.469 *p*_adj_ = 0.495	−0.002 *p* = 0.495 *p*_adj_ = 0.495	−0.199 *p* = 0.207 *p*_adj_ = 0.345

CC: corpus callosum; WM: white matter; IV: intravenous; IT: intrathecal. * Correlation is significant at the 0.05 level (One-tailed). ** Correlation is significant at the 0.01 level (One-tailed). Note: *p*-values adjusted for FDR separately for each dependent variable (volumes and MTR means). *p*_adj_: FDR adjusted *p*-values.

**Table 5:** The relationship between age at diagnosis, sex, CRT, IT-MTX cumulative dose, and whole brain mean MTR.

	B	β	R	R^2^	ΔR^2^	F	ΔF	t
Step 1			0.422	0.178	0.178	1.623 (*p* = 0.194)	1.623 (*p* = 0.194)	
Current age	0.000	−0.187						−0.806 (*p* = 0.427)
Age at diagnosis	0.001	0.329						1.561 (*p* = 0.129)
Sex	−0.003	−0.129						−0.710 (*p* = 0.483)
Cranial radiotherapy	0.008	0.281						1.634 (*p* = 0.113)
Step 2			0.585	0.342	0.164	3.018 (*p* = 0.026)	7.244 (*p* = 0.012)	
Current age	−0.001	−0.316						−1.463 (*p* = 0.154)
Age at diagnosis	0.000	−0.136						−0.526 (*p* = 0.603)
Sex	−0.005	−0.200						−1.192 (*p* = 0.243)
Cranial radiotherapy	0.007	0.253						1.619 (*p* = 0.116)
IT-MTX dose	0.000	−0.704						−2.692 (*p* = 0.012)
Step 3			0.585	0.342	0.000	2.428 (*p* = 0.051)	0.001 (*p* = 0.974)	
Current age	−0.001	−0.316						−1.435 (*p* = 0.162)
Age at diagnosis	0.000	−0.135						−0.508 (*p* = 0.615)
Sex	−0.005	−0.202						−1.094 (*p* = 0.283)
Cranial radiotherapy	0.007	0.254						1.584 (*p* = 0.124)
IT-MTX dose	0.000	−0.702						−2.586 (*p* = 0.015)
Leucovorin dose	0.000	−0.006						−0.033 (p = 0.974)
Step 4			0.585	0.343	0.000	1.694 (*p* = 0.147)	0.008 (*p* = 0.992)	
Current age	−0.001	−0.306						−1.103 (*p* = 0.280)
Age at diagnosis	0.000	−0.111						−0.295 (*p* = 0.770)
Sex	−0.005	−0.203						−1.059 (*p* = 0.299)
Cranial radiotherapy	0.007	0.261						1.337 (*p* = 0.193)
IT-MTX dose	0.000	−0.692						−2.001 (*p* = 0.056)
Leucovorin dose	0.000	−0.010						−0.051 (*p* = 0.959)
IT-MTX dose × sex	0.000	0.011						0.041 (*p* = 0.968)
IT-MTX dose × age at diagnosis	0.000	0.026						0.101 (*p* = 0.921)

B: beta; β: standardized beta; Δ: variation.

## Data Availability

The data presented in this study are available on request from the corresponding author.

## Supplementary Materials

The following supporting information can be downloaded at: https://www.mdpi.com/article/10.3390/cancers16061208/s1, Table S1: Brain volume outcomes (mm^3^); Table S2: Magnetization transfer imaging outcomes (MTR means); Table S3: Pearson’s *r* for directional correlations conducted between neuroimaging outcomes and neuropsychological indices; Table S4: The relationship between age at diagnosis, sex, CRT, IT-MTX cumulative dose, and corpus callosum mean MTR; Table S5: The relationship between age at diagnosis, sex, CRT, IT-cytarabine cumulative dose, and whole brain mean MTR; Table S6: The relationship between age at diagnosis, sex, CRT, IT-cytarabine cumulative dose, and corpus callosum mean MTR; Table S7: The relationship between age at diagnosis, sex, CRT, IT-hydrocortisone cumulative dose, and whole brain mean MTR; Table S8: The relationship between age at diagnosis, sex, CRT, IT-hydrocortisone cumulative dose, and corpus callosum mean MTR.
