# Supplementary material for: Cumulative Dosage of Intrathecal Chemotherapy Agents Predicts White Matter Integrity in Long-Term Survivors of Acute Lymphoblastic Leukemia: A PETALE Study
Source: Cancers (Basel). 2024 Mar 19;16(6):1208. doi: 10.3390/cancers16061208 (PMC10969288; doi:10.3390/cancers16061208)
Supplement: Supplementary file 1 [file cancers-16-01208-s001.zip › cancers-2891428-supplementary.pdf]

Supplementary Materials

Table S1. Brain volume outcomes (mm^3).

	ALLs (n = 35) Mean (SD)	Controls (n = 21) Mean (SD)	p	FDR adj.-p	Effect Size r
WM volume	412,671.988 (55,115.5779)	442,292.971 (61,429.4994)	**0.034**	0.057	0.25
Intracranial vol.	1,339,179.48 (150,168.331)	1,413,861.25 (149,162.932)	**0.038**	0.057	0.24
WM vol. fraction	0.30795248 (0.02001171)	0.3122635 (0.01997215)	0.219	0.219	0.11

WM: White matter; vol.: volume. Values in bold where $p < 0.05$ (One-tailed).

Table S2. Magnetization transfer imaging outcomes (MTR means).

	ALLs (n = 35)	Controls (n = 21)	p	FDR adj.-p	Effect Size r
Whole brain	0.5760 (0.0120) [a]	0.5817 (0.0113) [a]	0.044 [c]	0.097	0.23
Right hemisphere	0.5777 (0.0130) [a]	0.5837 (0.0112) [a]	0.044 [c]	0.097	0.23
Left hemisphere	0.5745 (0.0116) [a]	0.5798 (0.0115) [a]	0.050 [c]	0.097	0.22
Corpus Callosum (CC)	0.5935 (0.0119) [a]	0.5976 (0.0125) [a]	0.117 [c]	0.176	0.16
Anterior CC	0.6024 (0.0134) [a]	0.6064 (0.0155) [a]	0.154 [c]	0.182	0.14
Mid-anterior CC	0.5859 [0.5611–0.6110] [b]	0.5911 (0.0143) [a]	0.182 [d]	0.182	0.12
Central CC	0.5740 [0.5297–0.6048] [b]	0.5766 (0.0218) [a]	0.054 [d]	0.097	0.22
Mid-posterior CC	0.5799 (0.0168) [a]	0.5872 (0.0144) [a]	0.051 [c]	0.097	0.22
Posterior CC	0.6040 (0.0105) [a]	0.6067 (0.0113) [a]	0.182 [c]	0.182	0.12

[a] Mean (Standard deviation); [b] Median [Range]; [c] Independent samples t-test; [d] Mann–Whitney U. Values in bold where $p \leq 0.05$ (One-tailed).

Table S3. Pearson's r for directional correlations conducted between neuroimaging outcomes and neuropsychological indices.

	FSIQ	GAI	VCI	PRI	WMI	PSI	EF Index
MTR means							
Whole brain	0.203<br>p = 0.067<br>p_{adj} = 0.115	0.227 *<br>p = 0.046<br>p_{adj} = 0.067	0.239 *<br>p = 0.038<br>p_{adj} = 0.057	0.141<br>p = 0.151<br>p_{adj} = 0.239	0.023<br>p = 0.432<br>p_{adj} = 0.498	0.200<br>p = 0.073<br>p_{adj} = 0.125	0.081<br>p = 0.277<br>p_{adj} = 0.409
Right hemisphere	0.234 *<br>p = 0.041<br>p_{adj} = 0.115	0.241 *<br>p = 0.037<br>p_{adj} = 0.067	0.240*<br>p = 0.037<br>p_{adj} = 0.057	0.160<br>p = 0.120<br>p_{adj} = 0.239	0.051<br>p = 0.355<br>p_{adj} = 0.498	0.260 *<br>p = 0.029<br>p_{adj} = 0.125	0.131<br>p = 0.168<br>p_{adj} = 0.409
Left hemisphere	0.159<br>p = 0.121<br>p_{adj} = 0.156	0.201<br>p = 0.069<br>p_{adj} = 0.078	0.228 *<br>p = 0.046<br>p_{adj} = 0.059	0.109<br>p = 0.212<br>p_{adj} = 0.239	−0.007<br>p = 0.480<br>p_{adj} = 0.498	0.130<br>p = 0.174<br>p_{adj} = 0.224	0.022<br>p = 0.437<br>p_{adj} = 0.437
Corpus callosum (CC)	0.215<br>p = 0.056<br>p_{adj} = 0.115	0.259 *<br>p = 0.027<br>p_{adj} = 0.067	0.271 *<br>p = 0.022<br>p_{adj} = 0.057	0.170<br>p = 0.105<br>p_{adj} = 0.239	0.052<br>p = 0.351<br>p_{adj} = 0.498	0.192<br>p = 0.083<br>p_{adj} = 0.125	0.065<br>p = 0.318<br>p_{adj} = 0.409
Anterior CC	0.193<br>p = 0.077<br>p_{adj} = 0.115	0.228 *<br>p = 0.045<br>p_{adj} = 0.451	0.212<br>p = 0.058<br>p_{adj} = 0.065	0.174<br>p = 0.100<br>p_{adj} = 0.239	0.022<br>p = 0.436<br>p_{adj} = 0.498	0.193<br>p = 0.081<br>p_{adj} = 0.125	0.091<br>p = 0.253<br>p_{adj} = 0.409
Mid-anterior CC	0.244 *<br>p = 0.035<br>p_{adj} = 0.115	0.282 *<br>p = 0.018<br>p_{adj} = 0.067	0.243 *<br>p = 0.035<br>p_{adj} = 0.057	0.249 *<br>p = 0.032<br>p_{adj} = 0.239	0.094<br>p = 0.246<br>p_{adj} = 0.498	0.214<br>p = 0.060<br>p_{adj} = 0.125	0.128<br>p = 0.174<br>p_{adj} = 0.409

Central CC	−0.022<br>p = 0.435<br>p_{adj} = 0.435	−0.017<br>p = 0.451<br>p_{adj} = 0.451	0.011<br>p = 0.468<br>p_{adj} = 0.468	−0.026<br>p = 0.426<br>p_{adj} = 0.426	−0.046<br>p = 0.370<br>p_{adj} = 0.498	0.005<br>p = 0.486<br>p_{adj} = 0.486	−0.073<br>p = 0.298<br>p_{adj} = 0.409
Mid-posterior CC	0.232 *<br>p = 0.043<br>p_{adj} = 0.115	0.238 *<br>p = 0.039<br>p_{adj} = 0.067	0.278 *<br>p = 0.019<br>p_{adj} = 0.057	0.123<br>p = 0.184<br>p_{adj} = 0.239	0.095<br>p = 0.244<br>p_{adj} = 0.498	0.194<br>p = 0.080<br>p_{adj} = 0.125	0.107<br>p = 0.215<br>p_{adj} = 0.409
Posterior CC	0.147<br>p = 0.139<br>p_{adj} = 0.156	0.219<br>p = 0.052<br>p_{adj} = 0.067	0.258 *<br>p = 0.027<br>p_{adj} = 0.057	0.111<br>p = 0.208<br>p_{adj} = 0.239	0.001<br>p = 0.498<br>p_{adj} = 0.498	0.115<br>p = 0.204<br>p_{adj} = 0.229	−0.044<br>p = 0.373<br>p_{adj} = 0.420
Volumes							
White matter (WM)	0.227 *<br>p = 0.046<br>p_{adj} = 0.108	0.368 **<br>p = 0.003<br>p_{adj} = 0.009	0.355 **<br>p = 0.004<br>p_{adj} = 0.012	0.246 *<br>p = 0.034<br>p_{adj} = 0.075	0.021<br>p = 0.439<br>p_{adj} = 0.439	0.067<br>p = 0.314<br>p_{adj} = 0.371	0.149<br>p = 0.137<br>p_{adj} = 0.199
Intracranial volume	0.192<br>p = 0.078<br>p_{adj} = 0.108	0.324 **<br>p = 0.007<br>p_{adj} = 0.011	0.311 **<br>p = 0.010<br>p_{adj} = 0.015	0.222 *<br>p = 0.050<br>p_{adj} = 0.075	−0.063<br>p = 0.321<br>p_{adj} = 0.439	0.074<br>p = 0.297<br>p_{adj} = 0.371	0.115<br>p = 0.199<br>p_{adj} = 0.199
WM volume fraction	0.168<br>p = 0.108<br>p_{adj} = 0.108	0.239 *<br>p = 0.038<br>p_{adj} = 0.038	0.232 *<br>p = 0.043<br>p_{adj} = 0.043	0.151<br>p = 0.133<br>p_{adj} = 0.133	0.152<br>p = 0.131<br>p_{adj} = 0.393	0.046<br>p = 0.371<br>p_{adj} = 0.371	0.130<br>p = 0.169<br>p_{adj} = 0.199

* Correlation is significant at the 0.05 level (One-tailed). ** Correlation is significant at the 0.01 level (1-tailed). Note: p-values adjusted for FDR separately for each dependent variable (neuropsychological indices) for MTRs and volumes separately. p_{adj}: FDR adjusted p-values.

Table S4. The relationship between age at diagnosis, sex, CRT, IT-MTX cumulative dose, and corpus callosum mean MTR.

	**B**	**β**	**R**	**R²**	**ΔR²**	**F**	**ΔF**	**t**
Step 1			0.534	0.285	0.285	2.987 (p = 0.035)	2.987 (p = 0.035)	
Current age	0.000	−0.093						−0.432 (p = 0.669)
Age at diagnosis	0.000	0.216						1.099 (p = 0.281)
Sex	−0.009	−0.366						−2.160 (p = 0.039)
Cranial radiotherapy	0.009	0.326						2.036 (p = 0.051)
Step 2			0.628	0.394	0.109	3.772 (p = 0.009)	5.228 (p = 0.030)	
Current age	0.000	−0.199						−0.959 (p = 0.345)
Age at diagnosis	0.000	−0.163						−0.658 (p = 0.516)
Sex	−0.010	−0.424						−2.636 (p = 0.013)
Cranial radiotherapy	0.008	0.304						2.024 (p = 0.052)
IT-MTX dose	0.000	−0.574						−2.286 (p = 0.030)
Step 3			0.628	0.394	0.000	3.040 (p = 0.020)	0.018 (p = 0.895)	
Current age	0.000	−0.198						−0.936 (p = 0.357)
Age at diagnosis	0.000	−0.159						−0.623 (p = 0.538)
Sex	−0.010	−0.433						−2.444 (p = 0.021)
Cranial radiotherapy	0.009	0.306						1.991 (p = 0.056)
IT-MTX dose	0.000	−0.567						−2.176 (p = 0.038)
Leucovorin dose	0.000	−0.022						−0.133 (p = 0.895)
Step 4			0.641	0.411	0.017	2.268 (p = 0.055)	0.366 (p = 0.697)	
Current age	0.000	−0.087						−0.332 (p = 0.743)

Age at diagnosis	−0.001	−0.305						−0.859 (p = 0.398)
Sex	−0.010	−0.434						−2.390 (p = 0.024)
Cranial radiotherapy	0.007	0.235						1.270 (p = 0.215)
IT-MTX dose	0.000	−0.718						−2.196 (p = 0.037)
Leucovorin dose	0.000	0.029						0.159 (p = 0.875)
IT-MTX dose * sex	0.000	0.204						0.796 (p = 0.433)
IT-MTX dose * age at diagnosis	0.000	−0.137						−0.554 (p = 0.585)

B: beta; β: standardized beta; Δ: variation.

Table S5. The relationship between age at diagnosis, sex, CRT, IT-cytarabine cumulative dose, and whole brain mean MTR.

	B	β	R	R^2	ΔR^2	F	ΔF	t
Step 1			0.422	0.178	0.178	1.623 (p = 0.194)	1.623 (p = 0.194)	
Current age	0.000	−0.187						−0.806 (p = 0.427)
Age at diagnosis	0.001	0.329						1.561 (p = 0.129)
Sex	−0.003	−0.129						−0.710 (p = 0.483)
Cranial radiotherapy	0.008	0.281						1.634 (p = 0.113)
Step 2			0.571	0.326	0.148	2.801 (p = 0.035)	6.353 (p = 0.017)	
Current age	−0.001	−0.284						−1.311 (p = 0.200)
Age at diagnosis	0.000	−0.026						−0.108 (p = 0.915)
Sex	−0.005	−0.186						−1.102 (p = 0.279)
Cranial radiotherapy	0.008	0.268						1.693 (p = 0.101)
IT-cytarabine dose	0.000	−0.582						−2.521 (p = 0.017)
Step 3			0.573	0.328	0.002	1.882 (p = 0.112)	0.046 (p = 0.956)	
Current age	−0.001	−0.278						−1.061 (p = 0.298)
Age at diagnosis	0.000	−0.096						−0.278 (p = 0.783)
Sex	−0.005	−0.194						−1.094 (p = 0.284)
Cranial radiotherapy	0.007	0.243						1.326 (p = 0.196)
IT-cytarabine dose	0.000	−0.652						−1.950 (p = 0.062)
IT-cytarabine dose * sex	0.000	0.054						0.209 (p = 0.836)
IT-cytarabine dose * age at diagnosis	0.000	−0.075						−0.272 (p = 0.788)

B: beta; β: standardized beta; Δ: variation.

Table S6. The relationship between age at diagnosis, sex, CRT, IT-cytarabine cumulative dose, and corpus callosum mean MTR.

	B	β	R	R^2	ΔR^2	F	ΔF	t
Step 1			0.534	0.285	0.285	2.987 (p = 0.035)	2.987 (p = 0.035)	
Current age	0.000	−0.093						−0.432 (p = 0.669)
Age at diagnosis	0.000	0.216						1.099 (p = 0.281)
Sex	−0.009	−0.366						−2.160 (p = 0.039)
Cranial radiotherapy	0.009	0.326						2.036 (p = 0.051)
Step 2			0.618	0.382	0.097	3.579 (p = 0.012)	4.536 (p = 0.042)	
Current age	0.000	−0.172						−0.830 (p = 0.414)
Age at diagnosis	0.000	−0.071						−0.310 (p = 0.759)
Sex	−0.010	−0.413						−2.549 (p = 0.016)
Cranial radiotherapy	0.009	0.316						2.085 (p = 0.046)

IT-cytarabine dose	0.000	−0.471						−2.130 (*p* = 0.042)
Step 3			0.623	0.389	0.007	2.451 (*p* = 0.044)	0.153 (*p* = 0.859)	
Current age	0.000	−0.118						−0.472 (*p* = 0.641)
Age at diagnosis	0.000	−0.143						−0.432 (*p* = 0.669)
Sex	−0.010	−0.428						−2.528 (*p* = 0.018)
Cranial radiotherapy	0.008	0.290						1.654 (*p* = 0.110)
IT-cytarabine dose	0.000	−0.576						−1.806 (*p* = 0.082)
IT-cytarabine dose * sex	0.000	0.137						0.551 (*p* = 0.586)
IT-cytarabine dose * age at diagnosis	0.000	−0.058						−0.220 (*p* = 0.828)

B: beta; β: standardized beta; Δ: variation.

Table S7. The relationship between age at diagnosis, sex, CRT, IT-hydrocortisone cumulative dose, and whole brain mean MTR.

	**B**	**β**	**R**	**R²**	**ΔR²**	**F**	**ΔF**	**t**
Step 1			0.422	0.178	0.178	1.623 (*p* = 0.194)	1.623 (*p* = 0.194)	
Current age	0.000	−0.187						−0.806 (*p* = 0.427)
Age at diagnosis	0.001	0.329						1.561 (*p* = 0.129)
Sex	−0.003	−0.129						−0.710 (*p* = 0.483)
Cranial radiotherapy	0.008	0.281						1.634 (*p* = 0.113)
Step 2			0.563	0.317	0.139	2.695 (*p* = 0.041)	5.917 (*p* = 0.021)	
Current age	−0.001	−0.557						−2.118 (*p* = 0.043)
Age at diagnosis	0.001	0.624						2.712 (*p* = 0.011)
Sex	−0.006	−0.261						−1.473 (*p* = 0.152)
Cranial radiotherapy	−0.004	−0.129						−0.556 (*p* = 0.582)
IT-hydrocortisone dose	0.000	−0.696						−2.433 (*p* = 0.021)
Step 3			0.565	0.320	0.002	1.812 (*p* = 0.126)	0.046 (*p* = 0.955)	
Current age	−0.001	−0.626						−1.593 (*p* = 0.123)
Age at diagnosis	0.001	0.650						2.564 (*p* = 0.016)
Sex	−0.008	−0.329						−0.505 (*p* = 0.618)
Cranial radiotherapy	−0.004	0.134						−0.435 (*p* = 0.667)
IT-hydrocortisone dose	0.000	−0.750						−2.062 (*p* = 0.049)
IT-hydrocortisone dose * sex	0.000	−0.071						−0.091 (*p* = 0.928)
IT-hydrocortisone dose * age at diagnosis	0.000	−0.060						−0.295 (*p* = 0.770)

B: beta; β: standardized beta; Δ: variation.

Table S8. The relationship between age at diagnosis, sex, CRT, IT-hydrocortisone cumulative dose, and corpus callosum mean MTR.

	**B**	**β**	**R**	**R²**	**ΔR²**	**F**	**ΔF**	**t**
Step 1			0.534	0.285	0.285	2.987 (*p* = 0.035)	2.987 (*p* = 0.035)	
Current age	0.000	−0.093						−0.432 (*p* = 0.669)
Age at diagnosis	0.000	0.216						1.099 (*p* = 0.281)
Sex	−0.009	−0.366						−2.160 (*p* = 0.039)
Cranial radiotherapy	0.009	0.326						2.036 (*p* = 0.051)
Step 2			0.625	0.390	0.105	3.713 (*p* = 0.010)	5.016 (*p* = 0.033)	
Current age	−0.001	−0.416						−1.672 (*p* = 0.105)

Age at diagnosis	0.001	0.472						2.174 (p = 0.038)
Sex	−0.011	−0.481						−2.874 (p = 0.008)
Cranial radiotherapy	−0.001	−0.030						−0.137 (p = 0.892)
IT-hydrocortisone dose	0.000	−0.606						−2.240 (p = 0.033)
Step 3			0.635	0.403	0.013	2.605 (p = 0.034)	0.291 (p = 0.750)	
Current age	−0.001	−0.447						−1.215 (p = 0.235)
Age at diagnosis	0.001	0.435						1.830 (p = 0.078)
Sex	−0.018	−0.774						−1.269 (p = 0.215)
Cranial radiotherapy	0.003	0.096						0.333 (p = 0.742)
IT-hydrocortisone dose	0.000	−0.473						−1.388 (p = 0.177)
IT-hydrocortisone dose * sex	0.000	−0.390						−0.535 (p = 0.597)
IT-hydrocortisone dose * age at diagnosis	0.000	0.099						0.519 (p = 0.608)

B: beta; β: standardized beta; Δ: variation.
